# Oral Health-Related Quality-of-Life According to Dental Caries Severity, Body Mass Index and Sociodemographic Indicators in Children with Special Health Care Needs

**DOI:** 10.3390/jcm10214811

**Published:** 2021-10-20

**Authors:** Rawan Rasheed Alwattban, Lama Saleh Alkhudhayr, Sanaa Najeh Al-Haj Ali, Ra’fat Ibrahim Farah

**Affiliations:** 1 Dental Intern, College of Dentistry, Qassim University, Qassim 51452, Saudi Arabia; Rawan.alwattban@gmail.com (R.R.A.); Lama.s.alkhudhair@gmail.com (L.S.A.); 2 Department of Orthodontic and Pediatric Dentistry, College of Dentistry, Qassim University, Qassim 51452, Saudi Arabia; 3 Department of Prosthetic Dental Sciences, College of Dentistry, Qassim University, Qassim 51452, Saudi Arabia; dr.rafat.farah@qudent.org or

**Keywords:** body mass index, children, dental caries, oral health, quality of life, special health care needs

## Abstract

This study aimed to assess the impact of dental caries’ severity, body mass index (BMI), and sociodemographic factors on oral health-related quality of life (OHRQoL) for special health care needs (SHCN) children and the suitability of their caregivers as proxies to determine OHRQoL. This cross-sectional study recruited 107 pairs of SHCN children and their caregivers and asked them to complete a questionnaire on sociodemographic issues as well as the Arabic version of the early childhood oral health impact scale (A-ECOHIS). This was followed by a dental examination. Dental caries was measured using the dmft/DMFT index, while caries’ severity was also determined. The children’s height and weight were measured, and BMI (kg/m^2^) was recorded. Data were analyzed statistically using *t*-test, one-way ANOVA, and Poisson regression models. Our results revealed that the A-ECOHIS score was 10.93, while the OHRQoL was affected in 95.3% of children. The most-reported item was ‘pain in the teeth, mouth, or jaws’ (48.7%). By regression analysis, caries-free children (Odds Ratio (OR): 0.650) or those who had moderate caries (OR: 0.551) were less likely to have a negative impact on their OHRQoL than those with severe caries. Additionally, those whose caregivers had a maximum primary education (OR: 0.656) or whose occupation was in the health sector (OR: 0.721) were less likely to have a negative impact on their OHRQoL. Those who were ≤ 6 years old (OR: 1.188) were more likely to have a negative impact. BMI did not have a significant impact on the OHRQoL of the children. Further, we detected a significant positive correlation between children’s dmft/DMFT scores and the A-ECOHIS scores reported by the mothers. Given these variables, which included dental caries’ severity, but not BMI, and caregivers’ education level and occupation, plus the child’s age group, we found a significant impact on the OHRQoL. However, we found that mothers were better proxies for their children’s OHRQoL.

## 1. Introduction

The American Academy of Pediatric Dentistry (AAPD) defines special health care needs (SHCN) as ‘any physical, developmental, mental, sensory, behavioral, cognitive, or emotional impairment that requires medical management, health care intervention, and/or use of specialized services, and may impose limitations in performing daily activities or substantial limitations in a major life activity’ [1]. Medically compromised children in this category were reported to suffer a greater risk of oral diseases throughout their lives [2], as well as unmet dental needs, particularly for those who are more medically complex, with an increased risk for systemic and internal family barriers in finding care, based on their medical diagnoses. Additionally, those children, along with the majority of SHCN children, cannot understand, assume responsibility for, or cooperate with preventive oral health practices [3]. Several studies reported poorer oral health status among SHCN children than healthy ones [4,5,6,7]. Few studies have linked dental caries with obesity in these children [8]. According to Lee et al. [4], the existence and type of SHCN have a decisive effect on oral health conditions.

Oral health-related quality of life (OHRQoL) is defined as a multidimensional construct that reflects comfort when eating, sleeping, engaging in social interactions, and self-esteem: that is, satisfaction with oral health [9]. Several studies assessed the impact of dental caries on OHRQoL of healthy preschool children, primarily using the early childhood oral health impact scale (ECOHIS) as the instrument [10,11,12,13,14,15]. This scale allows caregivers to answer the questions on behalf of the children, as they lack the necessary cognitive skills to assess them on their own [16]. According to Lee et al. [17], the ECOHIS was more sensitive than other scales in assessing the impact of dental caries on OHRQoL of preschool children; however, few concerns were raised about the suitability of fathers as proxy assessors of their children’s OHRQoL compared to mothers [12,18]. Recently, Nora et al. [19] highlighted the negative impact of dental caries on the OHRQoL of preschool children, which tends to coincide with increasing caries’ severity. The impact of dental caries’ severity on the OHRQoL of SHCN children was not sufficiently investigated [6,20,21], perhaps with limited understanding of what was being evaluated [20].

In Saudi Arabia, a high prevalence of dental caries (77–79%) was reported among intellectually disabled and medically-compromised children [5,7,18,22]. Pani et al. [23] found that autistic children had reduced OHRQoL, although the impact of dental caries’ severity on their OHRQoL was not assessed. To date, the impact of different sociodemographic characteristics or the BMI on the OHRQoL of SHCN children is unclear. The suitability of caregivers as proxy assessors in determining the OHRQoL of SHCN children must be investigated. This is particularly significant in SHCN children when many are incapable of providing an assessment of their OHRQoL, have communication problems, or depend on their caregivers for oral and general health [23].

Thus, the aim of this study was: (1) to assess the impact of dental caries’ severity, BMI, and sociodemographic factors on OHRQoL in a subpopulation of children with SHCN; and (2) to assess the suitability of caregivers as proxies to determine the OHRQoL of SHCN children.

## 2. Methods

### 2.1. Study Population

This was a cross-sectional study conducted on a convenience sample of 107 SHCN children and their caregivers who attended the dental clinics of Buraydah maternity and children hospital, Qassim, Saudi Arabia, during the period between February and April 2021. Ethical approval was obtained from the College of Dentistry/Qassim University before the study was conducted (reference #: EA/6061/2021).

The inclusion criteria for the study were:

(1) Children 4 to 12 years of age;
(2) Cooperative children who allow dental examination;
(3) Children with a diagnosis, confirmed from the children’s medical records, of behavioral (e.g., anxiety, attention deficit hyperactivity disorder, autism spectrum disorder), congenital (e.g., Trisomy 21, congenital heart disease), developmental (e.g., cerebral palsy), or cognitive (e.g., intellectual disability) disorders or systemic diseases (e.g., childhood cancer, sickle cell disease) [2];
(4) Caregivers who speak and understand the Arabic language and live in Saudi Arabia;
(5) Caregivers who were willing to participate in the study and sign a written informed consent.

The exclusion criteria were:

(1) Individuals older than 12 years of age;
(2) Healthy children;
(3) Caregivers and their children who do not speak the Arabic language or reside in Saudi Arabia;
(4) Caregivers who refuse to participate in the study and sign a written informed consent.

Caregivers in the current study were either the mothers or the fathers of the children. Both were the legal guardians of the children.

### 2.2. Study Measures

Caregivers of the children were requested to answer a questionnaire consisting of two parts through a face-to-face interview. The first part comprised sociodemographic questions, while the second part comprised the Arabic version of the ECOHIS. This scale contains 13 questions corresponding to 4 descriptive domains for items included in the child impact section: symptoms, function, psychological, and self-image and social interaction, and 2 domains in the family impact section: parental distress and family function [22]. Response categories are scored from 0 = never, 1 = hardly ever; 2 = occasionally; 3 = often; 4 = very often; to 5 = do not know. The total score ranges between 0 and 52 [16,24]. The sociodemographic questions included the caregiver’s gender, caregivers’ education level, and occupation as well as the monthly family income, the child’s gender and age group, and an overall rating of the child’s dental health. Before interviewing the caregivers, the questionnaire was piloted on a sample of 10 caregivers, and all questions were clear.

### 2.3. Dental Examination

Following the completion of the caregivers’ questionnaires, dental examination of the children was carried out by one examiner using the daylight, disposable mirror, and explorer. Intra-examiner reliability was verified on a sample of 10 children with a kappa score exceeding 0.9. Dental caries was measured using the decayed (d), missing (m), and filled (f) teeth (dmft) index for the deciduous teeth and the Decayed (D), Missing (M), and Filled (F) teeth (DMFT) index for permanent according to the WHO criteria [25]. Both dmft/DMFT scores were recorded, separately when applicable, and combined, when applicable, by the sum of d +m + f + D + M + F according to the child’s age (≤6 or >6 years) in a similar manner to that adopted by Akhter et al. [6]. A score above null indicates the presence of dental caries, whereas a null score indicates the absence of dental caries [25]. Dental caries severity was then classified according to the number of untreated dental caries (0 free of caries; 1–2 moderate; and ≥3 high), where the upper cutoff values corresponded to the Significant Caries Index [26]. The height and weight of each child were also measured, and the BMI (kg/m^2^) was calculated based on the updated BMI-for age and gender percentile charts of the AAPD [27], and classified into one of four categories: underweight, healthy, overweight, or obese [28].

### 2.4. Data Analysis

Data were statistically analyzed using the SPSS computer software (Statistical Package for the Social Sciences Version 22, Chicago, IL, USA). Frequencies and percentages of the responses toward each question were generated. They were also calculated for the child’s dental health, dental caries status, dental caries severity, BMI, and health condition categories. Because the data were normally distributed, a *t*-test was used to compare means according to the child’s dental health and dental caries status, while one-way ANOVA and Tukey post hoc tests were used to compare means and determine the impact of dental caries severity and BMI on the A-ECOHIS score as well as child and family impact sections’ scores. Poisson regression modeling was used to confirm the association of the different independent variables with the A-ECOHIS score as well as the child and family impact sections scores. Further, the relationship between the dmft/DMFT score of the children and the A-ECOHIS score, as well as the score reported by each caregiver (farther vs. mother), was determined using Spearman’s correlation coefficient. Probability values of *p* <0.05 were considered statistically significant.

## 3. Results

The sociodemographic characteristics, characteristics of the child, and clinical examination findings are summarized in Table 1. The great majority of children (41.1%) had malignancy (either acute or in remission). Dental caries was present in 93.5% of the children, and 81.3% of those had severe caries. The dmft/DMFT score of the children was 7.11± 5.10. The greatest score was for children with congenital heart disease (9.52 ± 6.15), followed by children with down syndrome (7.00 ± 5.00), while the lowest score was for children with bleeding disorders (5.77 ± 3.91). With regards to BMI, underweight children had the greatest dmft/DMFT scores (8.90 ± 6.19), while obese children had the lowest scores (2.92 ± 2.43). SHCN children whose caregivers rated their dental health as poor had greater dmft/DMFT scores (9.45 ± 3.02) than those whose caregivers rated their dental health as good (6.84 ± 5.22).

Table 2 shows the responses to the A-ECOHIS items. In the child impact section, ‘pain in the teeth, mouth or jaws’ was the most frequently reported symptom (48.7%), while ‘feeling upset’ (36.4%) was the most frequently reported in the family impact section. Table 3 provides the descriptive statistics of the A-ECOHIS responses, ranges, floor effect, and comparisons according to dental caries severity. No impacts were reported by 8.4% of the caregivers in the child impact section and 15.9% in the family impact section. For overall A-ECOHIS, only 4.7% reported no impact. The score achieved in the child impact section (7.09) was higher than the family impact section (3.84). In the child impact section, the child function domain had the highest mean score (3.03). In the family impact section, the parental distress domain had a higher mean than the family function domain (2.24 vs. 1.59, respectively). Overall, the A-ECOHIS score from fathers’ reports was 11.13 ± 6.10 compared to 10.78 ± 7.01 from mothers’ reports.

Regarding the effect of dental caries severity, children with severe caries, in particular, had the highest mean scores in all domains of the scale, as well as each section separately. A statistically significant difference in mean scores between children with different caries severity was evident in the child symptoms domain, the child function domain, as well as the child impact section. Additionally, a statistically significant difference in mean scores was observed in the parental distress domain, the family impact section, as well as A-ECOHIS score (*p* < 0.05).

**Figure 1:**
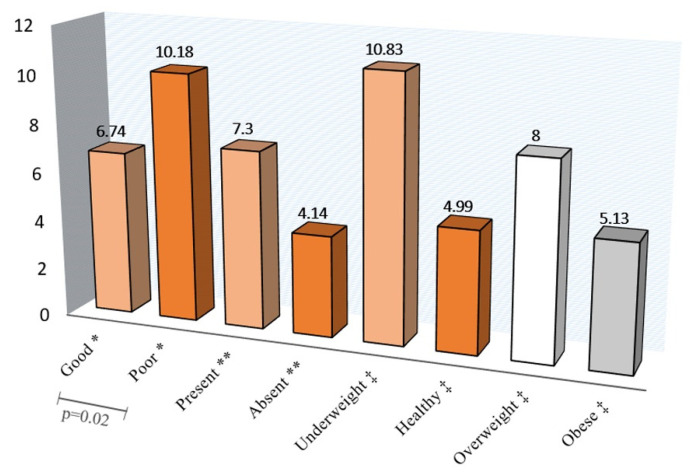
Child impact section score according to the * Child’s dental health, ** Dental caries status, and ‡ Body mass index.

**Figure 2:**
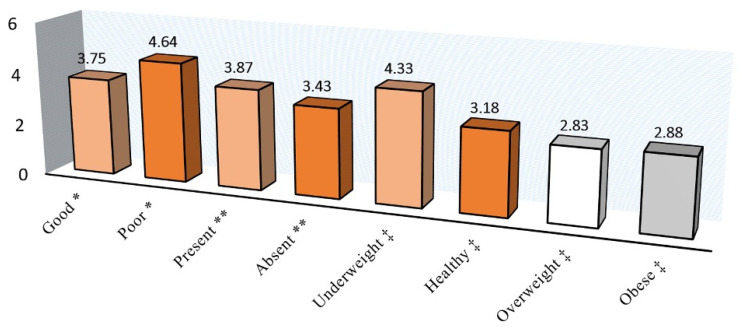
Family impact section score according to the * Child’s dental health, ** Dental caries status, and ‡ Body mass index.

**Figure 3:**
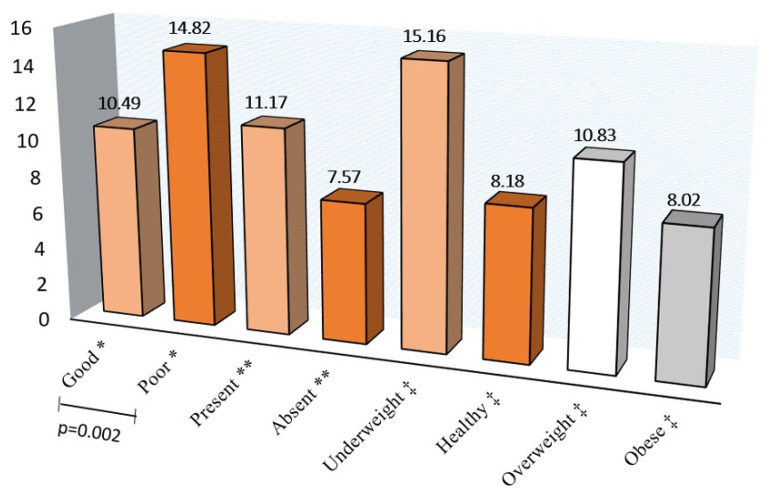
The A-ECOHIS score according to the * Child’s dental health, ** Dental caries status, and ‡ Body mass index.

Figure 1, Figure 2 and Figure 3 show the A-ECOHIS score, as well as individual child and family impact section scores, according to the child’s dental health, dental caries status, and BMI of the children. The child’s dental health had a significant impact on the child impact section score and the overall scale score, while BMI did not have a significant impact.

In the final multivariate model, dental caries severity, child dental health, child age group, and caregiver’s education level and occupation were found to impact the OHRQoL significantly (Table 4). Children whose caregiver had no schooling or a primary education level had 1.524 times fewer odds to score higher on the whole scale compared to those whose caregiver had a university degree (95% confidence interval (CI): 0.523–0.822). Additionally, those whose caregiver’s occupation was in the health sector had 1.386 times fewer odds to score higher than those who had a non-working caregiver (95% CI: 0.539–0.964). On the other hand, children who were ≤6 years old had 1.188 times higher odds to score higher than older children (95% CI: 1.028–1.373). Moreover, those who had a good dental health rating by their caregiver had 1.45 times fewer odds to score higher than those with a poor rating (95% CI: 0.574–0.828). Finally, caries-free children had 1.538 times fewer odds to score higher than those with severe caries (95% CI: 0.482–0.876), and those with moderate caries had 1.814 times fewer odds to score higher than those with severe caries (95% CI: 0.482–0.876).

**Figure 4:**
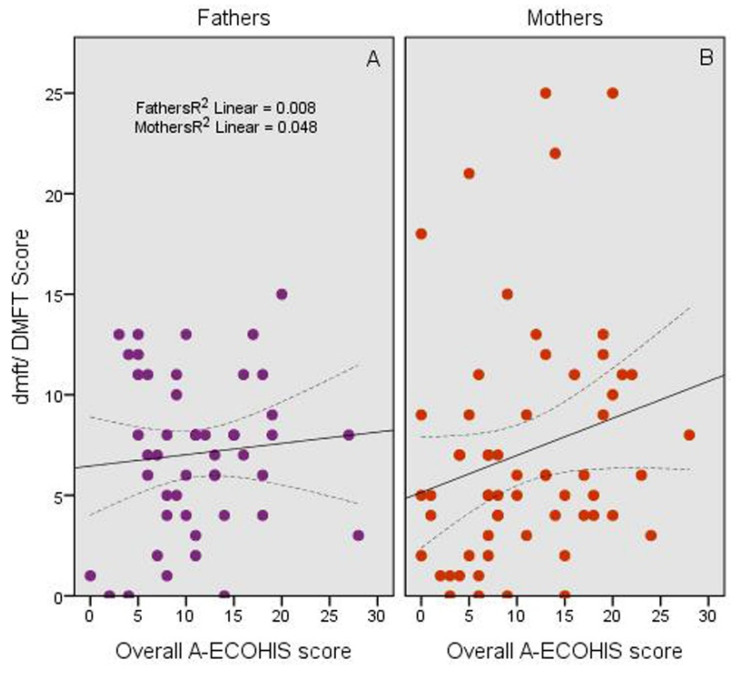
Relationship between the dmft/DMFT scores of the children and the A-ECOHIS score from reports of (**A**) fathers and (**B**) mothers. The middle lines show the line of best fit for the correlation and the two lateral lines show the 95% confidence intervals for the mean values.

With regards to the association of the caries experience scores of the children and the A-ECOHIS score, there was a significant positive correlation (*p* = 0.03), particularly with the score reported by the mothers (*p* = 0.032) (Figure 4).

## 4. Discussion

This study is the first to assess the impact of dental caries severity, sociodemographic variables, and BMI on the OHRQoL of a subpopulation of SHCN children and to assess the suitability of caregivers (parents) as proxy assessors of the OHRQoL of SHCN children. The A-ECOHIS, which was validated in Saudi Arabia, was chosen as the study instrument due to the lack of instruments directed to evaluate the OHRQoL of SHCN children and age group under study [29], particularly in the Arabic language, and, further, to enable comparisons with previous studies which used the same scale either in English or a different language [20,21,23]. Adopting a scale that has been validated in the same country is a strong point as differences in semantics and norms are not expected [30].

In the current study, the dmft/DMFT score of the children was 7.11, with 93.5% of them having dental caries despite that all children had health insurance coverage. These findings are higher than those reported among SHCN children in Brazil (68.75%) [20], KSA (56.7%) [8], and Bangladesh (55.6%) [6]. However, they are not surprising when the prevalent nature of dental caries in Saudi Arabia is considered among SHCN and healthy children (27.4–79%) [5,7,18,22,31,32]. Additionally, the present study recruited a hospital-based sample of children who were seeking treatment of pain from self-recognized oral health problems, and consequently, they were expected to have poor oral health [14,24,33]. Furthermore, many of the children were taking sugar-sweetened liquid oral medicines regularly or were suffering from hyposalivation (mainly those suffering malignancies) caused by their drugs, the treatment, or the disease process itself [5]. Children with congenital heart disease, followed by those with down syndrome, had the highest dmft/DMFT scores. Storhaug [34] and Brown [5] also found that children with congenital heart disease had high dmft scores or unmet dental treatment needs compared to several disabling conditions. However, the health condition in the present study did not have a significant impact on the OHRQoL of the children, although it had an impact on the child.

In the present study, the OHRQoL was affected in 95.3% of the children, which is greater than the percentage reported by Faker et al. [20] (68.75%), and Aggrawal et al. [21] (54%). The reported A-ECOHIS score in the present study (11.85) was greater than that in the above-mentioned studies (7.10) and (1.38). These findings are not surprising considering the greater caries experience in our population, as having more decayed teeth results in greater pain and consequently more impact on the quality of life. Another explanation would be that the children in the current study neither had problems in their verbal ability nor had an intellectual disability; hence, they should be able to express their feelings of pain or discomfort and the reasons for being upset to their caregivers very well [7].

The most frequently reported impact in the present study concerned the oral symptoms domain ‘pain in the teeth, mouth, or jaws’. In the family impact section, the ‘been upset’ item was the most reported one, and overall, negative impacts were greater on the child than on the family. Additionally, heavy floor effects were not observed. These results are consistent with previous studies on SHCN and healthy children from Saudi Arabia and elsewhere [6,11,20,24].

In the present study, the child’s dental health, as well as dental caries severity, negatively impacted the OHRQoL, whereas BMI did not have a significant impact. In particular, the child symptoms and function domains and the parental distress domain were affected by caries severity. Children who had either moderate or severe caries were more likely to have worse OHRQoL compared to those without dental caries. These findings seem common among SHCN and healthy children [10,11,12,19,20,21,23,24,33,35]. On the other hand, the finding concerning the lack of impact of BMI on OHRQoL perhaps stems from the controversial relation between weight and oral health. According to Alshehri et al. [36], conflicting results of BMI and dental caries in children were reported where some studies reported a positive association, while others reported a negative or no association.

The sociodemographic characteristics, including a family’s socioeconomic status, which may be evaluated by income and education, can affect the caregivers’ perceptions regarding their child’s oral health and is related to oral health conditions [24]. In the present study, the family monthly income did not have an impact on the OHRQoL of the children, unlike previous studies [35,37]. The relationship between sociodemographic characteristics and OHRQoL is not clear-cut [21]. In the present study, the caregiver’s education level and occupation and the child’s age group affected the OHRQoL of the children, where children whose caregiver had no schooling or a primary education level had better OHRQoL compared to those whose caregiver had a university degree. Additionally, those whose caregiver’s occupation was in the health sector had better OHRQoL than those with a non-working caregiver. The finding concerning the impact of the caregiver’s education level agrees with Chaffee et al. [33], who found that ECOHIS scores were lower when reported by caregivers of less educational attainment. They attributed that to the fact that quality-of-life measures are subjective in nature and may reflect the expectations of individuals who have adapted to a particular life situation, so it is probable that caregivers with a lower educational level are viewing caries as an unavoidable disease, which cannot be controlled, perhaps contributing to cognitive dissonance between caregivers’ quality-of-life perceptions and actual experiences. On the other hand, our finding of the impact of the caregiver’s occupation sector may be explained by the likelihood that caregivers working in the health sector judge differently due to the nature of their work and the emotional stress level than non-working caregivers who are at home all day focusing on the children. In previous studies on SHCN children using the ECOHIS or a different scale [6,20,23,28,29], the parents were the proxies to determine the OHRQoL of their children, regardless of their age, as many of the children, particularly those with intellectual disability, may not be enrolled in schools, or have communication problems, and consequently, their ability to read, comprehend, and answer the questions reliably, regardless of their age, is expected to be low [30]. In our study, the dmft/DMFT scores of the children correlated positively with the A-ECOHIS score. This finding perhaps suggests that the parents, or one of them, maybe opted as a proxy assessor of the OHRQoL of the SHCN child. As such, the reporting parent’s gender did not have a significant impact on the OHRQoL of the child; however, the finding that higher A-ECOHIS scores from mothers’ reports were translated into higher dmft/DMFT scores in their children, while those obtained from fathers did not support previous observations [12,18] that fathers may be inept proxy assessors for the OHRQoL but this time of their SHCN child, compared to mothers who are commonly perceived as the primary caregivers of the children [18]. However, what seemed concerning in the present study is the inaccurate perception of parents of their SHCN children’s dental health as the great majority of the parents (90%) rated their children’s dental health as good when caries experience scores of the children were not low. While, it seemed logical that children of caregivers who rated their children’s dental health as poor had higher dmft/DMFT scores as well were more likely to have a negative impact on their OHRQoL than those of caregivers who rated their children’s dental health as good, the finding that those represented a small proportion (10%) needs to be emphasized. This highlights a need for a reliable scale that can be used in the future to assess the degree of dependence of SHCN children, at least those without intellectual problems, to avoid the need for proxy assessors to determine their own OHRQoL. In the meantime, mothers could be selected as the proxy assessor of their SHCN children.

The present study has limitations; first, the design of the study is cross-sectional; hence, causal relationships cannot be established. Second, we have used a convenience sample of children and their caregivers from a single hospital; consequently, we do not claim our study to be representative of all SHCN children in the country. Additionally, it is impossible to reflect a wide range of SHCN categories using convenience and relatively small samples; therefore, larger samples stratified by broad SHCN categories will be needed for a more precise analysis. Furthermore, since the children presented seeking dental care, it could be suggested that they have worse OHRQoL than those who do not seek dental care, which may contribute to an overestimation in our results [35]. On the other hand, different studies have assessed the OHRQoL of SHCN and healthy children with convenience samples in hospitals or university institutions [20,28,29,38,39]. Therefore, even though our study is limited to extrapolate the results to the general population, it is relevant to infer associations to SHCN children attending dental services. Future studies should focus on finding a scale to assess the degree of dependence of SHCN children, as it would be best if those children, particularly the ones without intellectual disability, determine their own OHRQoL without the need for proxy assessors.

## 5. Conclusions

Dental caries’ severity, caregivers’ education level and occupation, and the child’s age group had a significant impact on the OHRQoL of the assessed subpopulation of SHCN children; however, BMI did not. Moreover, mothers were better proxies for their SHCN children’s OHRQoL.

## Figures and Tables

**Table 1:** Sociodemographic characteristics, characteristics of the child, and clinical examination findings.

Variable	Category	*N*	%
Sociodemographic factors			
Caregiver gender	Male	47	43.9
	Female	60	56.1
Caregiver education level	Never been to school or primary	16	15.0
	Intermediate or secondary	36	33.6
	University	55	51.4
Spouse education level	Never been to school or primary	14	13.1
	Intermediate or secondary	41	38.3
	University	52	48.6
Caregiver occupation	Education sector	32	29.9
	Health sector	9	8.4
	Other than the health and education sector	18	16.8
	Unemployed	48	44.9
Spouse occupation	Education sector	22	20.6
	Health sector	9	8.4
	Other than the health and education sector	40	37.4
	Unemployed	36	33.6
Family income	≤3200 USD	54	50.5
	>3200 USD	53	49.5
Characteristics of the child			
Child gender	Boy	62	57.9
	Girl	45	42.1
Child age group	≤6 years	25	23.4
	>6 years	82	76.6
Health condition	Bleeding disorders or coagulopathies	31	29
	Congenital heart disease	27	25.2
	Malignancy (acute or in remission)	44	41.1
	Down syndrome	5	4.7
Child dental health	Good	96	89.7
	Poor	11	10.3
Clinical examination findings			
Dental caries status	Present	100	93.5
	Absent	7	6.5
Dental caries severity	Caries free	7	6.5
	Moderate	13	12.1
	High	87	81.3
BMI	Underweight	10	9.3
	Healthy	76	71
	Overweight	9	8.4
	Obese	12	11.2

**Table 2:** Distribution of A-ECOHIS responses (*n* = 107).

		Never/Hardly Ever *N* (%)	Occasionally, Often, Very Often *N* (%)	Do not Know *N* (%)
Symptoms *	Pain	54 (50.5)	52 (48.7)	1 (0.9)
Function *	Difficulty drinking hot or cold beverages	75 (70.1)	27 (25.2)	5 (4.7)
	Difficulty eating	86 (80.4)	20 (18.7)	1 (0.9)
	Pronunciation difficulty	91 (85)	16 (14.9)	0 (0)
	Missed school or daycare	88 (82.2)	17 (15.9)	2 (1.9)
Psychology *	Trouble sleeping	92 (86)	15(14)	0 (0)
	Irritability or frustration	75 (70.1)	28 (26.1)	4 (3.7)
Self-image and social interaction *	Avoid smiling or laughing	92 (86)	15 (14)	0 (0)
	Avoid talking	92 (86)	15 (14)	0 (0)
Parental distress **	Been upset	67 (62.6)	39 (36.4)	1 (0.9)
	Felt guilty about child’s oral health	72 (67.3)	34 (30.9)	2 (1.9)
Family function **	Taken time off work	84 (78.5)	19 (17.8)	4 (3.7)
	Financial impact	87 (81.4)	18 (16.9)	2 (1.9)

*: Child impact section, ** Family impact section; A-ECOHIS: Arabic version of the Early Childhood Oral Health Impact Scale.

**Table 3:** Descriptive distributions of the A-ECOHIS responses as well as caries severity for different domains and the overall scale.

Variables	Items (*N*)	Possible Range	Floor Effect *N* (% Score Zero)	Mean (SD)	Caries Severity			*p*-Value
					Caries Free ^1^	Moderate ^2^	High ^3^	
**Child impact section**	9	0–36	9 (8.4)	7.09 (4.66)	4.14 (3.85)	4.85 (4.39)	7.67 (4.62)	0.027 * (2 vs. 3)
Child symptoms domain	1	0–4	20 (18.7)	1.42 (0.97)	0.86 (0.90)	0.85 (.899)	1.55 (.949)	0.013 * (2 vs. 3)
Child function domain	4	0–16	24 (22.42)	3.03 (2.68)	1.2857 (1.89)	1.6923 (2.097)	3.3793 (2.72)	0.020 *
Child psychology domain	2	0–8	35 (32.71)	1.64 (1.56)	1.1429 (1.21499)	1.6154 (1.66024)	1.6897 (1.57974)	0.675
Child self-image and social interaction domain	2	0–8	60 (56)	0.99 (1.46)	0.8571 (1.06)	0.6923 (1.03)	1.0460 (1.55)	0.703
**Family impact section**	4	0–16	17 (15.9)	3.84 (3.31)	3.43 (3.86)	1.77 (2.20)	4.18 (3.31)	0.045 * (2 vs. 3)
Parental distress domain	2	0–8	31 (28.97)	2.24 (2.28)	1.8571 (2.41)	0.7692 (1.23)	2.4943 (2.32)	0.034 * (2 vs. 3)
Family function domain	2	0–8	38 (35.51)	1.59 (1.81)	1.5714 (1.90)	1.0000 (1.47)	1.6897 (1.85)	0.444
A-ECOHIS items	13	0–52	5 (4.7)	10.93 (6.59)	7.57 (5.25)	6.62 (5.42)	11.85 (6.56)	0.010 * (2 vs 3)

A-ECOHIS: Arabic version of the Early Childhood Oral Health Impact Scale, * statistically significant. 1 for Caries free, 2 for moderate caries, and 3 for high caries.

**Table 4:** Variables associated with A-ECOHIS score, child impact, and family impact section scores.

Variables	Category	CIS	FIS	Overall Scale
		Odds Ratio (95% Confidence Interval)		
Caregiver gender	Male compared to female ^reference^	1.280 (1.070–1.532) †	0.629 (0.488–0.809) ‡	1.010 (0.873–1.167)
Caregiver education level	No schooling or primary	0.652 (0.493–0.863) †	0.665 (0.452–0.979) *	0.656 (0.523–0.822) ‡
	Intermediate or secondary	1.073 (0.885–1.301)	1.018 (0.780–1.328)	1.046 (0.895–1.221)
	University ^reference^	1	1	1
Spouse education level	No schooling or primary	0.756 (0.562–1.018)	1.046 (0.718–1.525)	0.856 (0.678–1.081)
	Intermediate or secondary	0.965 (0.801–1.162)	0.806 (0.621–1.045)	0.906 (0.779–1.054)
	University ^reference^	1	1	1
Caregiver occupation	Education sector	0.836 (0.669–1.044)	0.987 (0.733–1.328)	0.869 (0.728–1.037)
	Health sector	0.743 (0.520–1.060)	0.714 (0.426–1.195)	0.721 (0.539–0.964) *
	Other	0.932 (0.733–1.184)	0.848 (0.590–1.220)	0.894 (0.732–1.091)
	Unemployed ^reference^	1	1	1
Spouse occupation	Education sector	1.031 (0.813–1.307)	0.901 (0.657–1.236)	0.994 (0.823–1.200)
	Health sector	1.050 (0.768–1.436)	1.403 (0.955–2.063)	1.177 (0.925–1.498)
	Other	1.243 (1.023–1.510) *	0.808 (0.614–1.065)	1.079 (0.921–1.264)
	Unemployed ^reference^	1	1	1
Family income	≤3200 USD compared to >3200 USD ^reference^	0.970 (0.805–1.168)	0.968 (0.747–1.253)	0.971 (0.836–1.129)
Child Gender	Boy compared to girl ^reference^	0.986 (0.832–1.168)	1.095 (0.870–1.379)	1.024 (0.894–1.174)
Child age group	≤6 compared to >6 years ^reference^	1.059 (0.883–1.271)	1.468 (1.153–1.870) †	1.188 (1.028–1.373)*
Child dental health	Good compared to poor ^reference^	0.581 (0.463–0.729) ‡	0.915 (0.665–1.258)	0.689 (0.574–0.828) ‡
Caries severity	Caries free	0.592 (0.398–0.881) *	0.704 (0.444–1.115)	0.650 (0.482–0.876) †
	Moderate	0.674 (0.509–0.894) †	0.366 (0.232–0.578) ‡	0.551 (0.434–0.700) ‡
	High ^reference^	1	1	1
Health condition	Bleeding disorders	0.626 (0.443–0.885) †	1.336 (0.711–2.510)	0.776 (0.575–1.048)
	Congenital heart disease	0.796 (0.571–1.111)	1.750 (0.945–3.239)	1.010 (0.756–1.351)
	Malignancy	0.601 (0.428–0.846) †	1.282 (0.691–2.379)	0.750 (0.558–1.007)
	Down syndrome ^reference^	1	1	1

* *p* < 0.05, † *p* < 0.01, and ‡ *p* < 0.001. CIS: child impact section. FIS: family impact section.

## Data Availability

The data used to support the findings of this study can be made available upon request to the corresponding author.
